# Complex Adaptive Immunity to Enteric Fevers in Humans: Lessons Learned and the Path Forward

**DOI:** 10.3389/fimmu.2014.00516

**Published:** 2014-10-27

**Authors:** Marcelo B. Sztein, Rosangela Salerno-Goncalves, Monica A. McArthur

**Affiliations:** ^1^Department of Pediatrics, Center for Vaccine Development (CVD), University of Maryland School of Medicine, Baltimore, MD, USA

**Keywords:** *Salmonella* Typhi, *Salmonella* Paratyphi, enteric fever, typhoid fever, human immunity, CMI, multifunctional T-cells, microbiota

## Abstract

*Salmonella enterica* serovar Typhi (*S*. Typhi), the causative agent of typhoid fever, and *S*. Paratyphi A and B, causative agents of paratyphoid fever, are major public health threats throughout the world. Although two licensed typhoid vaccines are currently available, they are only moderately protective and immunogenic necessitating the development of novel vaccines. A major obstacle in the development of improved typhoid, as well as paratyphoid vaccines is the lack of known immunological correlates of protection in humans. Considerable progress has been made in recent years in understanding the complex adaptive host responses against *S*. Typhi. Although the induction of *S*. Typhi-specific antibodies (including their functional properties) and memory B cells, as well as their cross-reactivity with *S*. Paratyphi A and *S*. Paratyphi B has been shown, the role of humoral immunity in protection remains undefined. Cell mediated immunity (CMI) is likely to play a dominant role in protection against enteric fever pathogens. Detailed measurements of CMI performed in volunteers immunized with attenuated strains of *S*. Typhi have shown, among others, the induction of lymphoproliferation, multifunctional type 1 cytokine production, and CD8^+^ cytotoxic T-cell responses. In addition to systemic responses, the local microenvironment of the gut is likely to be of paramount importance in protection from these infections. In this review, we will critically assess current knowledge regarding the role of CMI and humoral immunity following natural *S*. Typhi and *S*. Paratyphi infections, experimental challenge, and immunization in humans. We will also address recent advances regarding cross-talk between the host’s gut microbiota and immunization with attenuated *S*. Typhi, mechanisms of systemic immune responses, and the homing potential of *S*. Typhi-specific B- and T-cells to the gut and other tissues.

## Introduction

Enteric fevers encompass typhoid fever caused by the Gram-negative intracellular bacterium *Salmonella enterica* serovar Typhi (*S*. Typhi) and paratyphoid fever caused largely by *S. enterica* serovars Paratyphi A and B (*S*. Paratyphi) (1, 2). Most cases of enteric fever are caused by *S*. Typhi (3). However, infections caused by *S*. Paratyphi A have been increasing in recent years, particularly in Asia (2, 4–7). Typhoid and paratyphoid fevers are life-threatening illnesses exhibiting very similar clinical features (2, 8). Humans are the only reservoir for these infections. The disease spreads by the fecal–oral route via contaminated food and water (9). In industrialized countries, enteric fevers are rare with most infections occurring in military personnel and in individuals traveling to endemic areas. According to the CDC, in the United States, it is estimated that ~5,700 cases of *S*. Typhi infection occur annually, mostly acquired while individuals are traveling internationally. However, *S*. Typhi and *S*. Paratyphi infections are a major public health problem in the developing world (9–13). It is estimated that 26.9 million new cases of typhoid fever occur annually with about 1% mortality (9–13). Based on data provided by the World Health Organization, 90% of these typhoid deaths occur in Asia, and most victims are children under 5 years of age (14). Furthermore, antimicrobial treatment of enteric fever and asymptomatic carriers has become increasingly complicated due to the emergence of multidrug-resistant strains of *S*. Typhi and *S*. Paratyphi A (7, 15, 16). Thus, there has been an increased emphasis on control measures, such as improved sanitation, food hygiene, and vaccination (8, 10, 17). It has also become evident that a better understanding of the host immune responses against *S*. Typhi and *S*. Paratyphi are required. This review will focus on the adaptive human immune responses [i.e., humoral and cell mediated immunity (CMI)] to *S*. Typhi and *S*. Paratyphi acquired through natural infection, experimental challenge, and vaccination. For discussions of the “mouse model of *Salmonella* infection,” the reader is referred to excellent reviews included in this “Frontiers in Immunology Research Topic” compilation.

## Immunity Elicited in Natural Infections Caused by *S*. Typhi and *S*. Paratyphi

*Salmonella* Typhi is a facultative intracellular bacterium that causes an acute generalized infection of the reticuloendothelial system (RES), intestinal lymphoid tissue, and gallbladder in humans (18). Classical symptoms include gradual onset of sustained fever, chills, hepatosplenomegaly, and abdominal pain. In some cases, patients experience rash, nausea, anorexia, diarrhea, or constipation, headache, relative bradycardia, and reduced level of consciousness (19). After *S*. Typhi ingestion, the period of incubation ranges from 3 to 21 days, with the mean incidence between 8 and 14 days (19). Without effective treatment, typhoid fever has a case-fatality rate of 10–30%. This number can be reduced to 1–4% with appropriate therapy (10). In addition, a small number of individuals become “carriers.” These individuals, after recovering from acute *S*. Typhi infection, keep shedding *S*. Typhi in their feces and are able to spread the disease.

After ingestion of contaminated food or water, sufficient numbers of *S*. Typhi might survive the low pH of the stomach and cross the intestinal epithelial monolayer through mechanisms that involve M cells, dendritic cells (DC), passage through enterocytes in endocytic vacuoles, and/or disruption of tight junctions (paracellular route) (20, 21). Once in the lamina propria, *S*. Typhi can spread systemically and trigger innate and adaptive host immune responses.

Most of our knowledge of adaptive host immune responses to *S*. Typhi natural infection originates from studies involving individuals living in typhoid endemic areas (21–23). Clinical studies indicate that the development of protective immunity after recovery from typhoid fever is possible but that the frequency of individuals able to mount protective immune responses is low (22, 23). *S*. Typhi infections in individuals living in endemic areas elicit the appearance of both humoral and CMI responses. Anti-*S*. Typhi-specific antibodies against lipopolysaccharide (LPS), H (flagellin), Vi (*S*. Typhi capsular polysaccharide; virulence factor), porins, and heat-shock proteins (e.g., GroEL), among others, have been well documented in the sera of acute and convalescent typhoid fever patients (24–31). In addition, the presence of anti-*S*. Typhi secretory IgA (SIgA) was also described in intestinal fluids of typhoid patients (32). Of note, high-anti-Vi IgG antibodies are present in a considerable proportion of chronic biliary *S*. Typhi carriers, particularly in endemic areas. The presence of functional antibodies against *S*. Typhi (e.g., bactericidal activity), which increase with age has also been reported in healthy residents of typhoid endemic areas (33). However, the role that antibodies play in protection remains elusive. For example, susceptibility to typhoid infection has been reported to occur despite the presence of elevated titers of antibodies against O, H, and other *S*. Typhi antigens (22, 23, 29, 34).

Clinical observations suggest that CMI, particularly cytokines, play an important role in host defense against *Salmonella* infection. For example, increased susceptibility to invasive *Salmonella* infections, caused largely by non-typhoidal *Salmonella*, as well as a few *S*. Typhi and *S*. Paratyphi cases, have been reported in individuals with immune deficiencies for interferon (IFN)-γ, interleukin (IL)-12, IL-23, and STAT1 receptors (35–38). Moreover, significant genetic associations were reported between susceptibility or resistance to typhoid fever and HLA-DR and HLA-DQ MHC and tumor necrosis factor (TNF)-α alleles in Vietnam residents (39). Of note, although the data is sparse, it has been reported that human immunodeficiency virus (HIV) positive patients in an endemic area are at significantly increased risk for infection with *S*. Typhi and *S*. Paratyphi (40). However, these results will need further confirmation as other studies have failed to observe this association (36).

The importance of CMI in the host’s response to *S*. Typhi has also been derived from early studies in acute and chronic carrier typhoid patients, which demonstrated the presence of specific CMI responses, including antigen-specific lymphoproliferation, leukocyte migration inhibition, and rosette-forming cells (32, 41– 46). Moreover, elevated serum levels of IFN-γ, IL-6, and TNF-α receptor (TNF-R) p55 and TNF-R p75 were reported in *S*. Typhi and *S*. Paratyphi A-infected patients in Nepal (47). Interestingly, in these studies higher values of IL-6 and soluble TNF-R p55 were related to poorer outcome. In another study, Keuter et al. showed that levels of the anti-inflammatory mediators IL-1 receptor antagonist (IL-1RA), soluble TNF-R (p55 and p75), and IL-8 were higher in the acute phase than in the convalescent phase (48). In contrast, the production capacity of pyrogenic cytokines (TNF, IL-6) was depressed in the acute phase of typhoid fever but was restored during the convalescent phase. Of note, no differences were observed between patients with complicated or uncomplicated disease courses. These observations have been extended by recent studies in Bangladeshi typhoid patients, which have shown the induction of specific T-cell responses [e.g., production of IFN-γ, IL-17, macrophage inflammatory protein (MIP)-1β, lymphoproliferation] to purified *S*. Typhi antigens using a novel high-throughput technique (49– 51). Concerning the cellular source of cytokines/chemokines, experiments using human PBMC from healthy subjects and Ty21a vaccinees have shown that, in addition to lymphocytes, stimulation with *S*. Typhi flagella induced the rapid *de novo* synthesis of TNF-α and IL-1β, followed by IL-6 and IL-10 in macrophages (52). Follow-up experiments indicated that whole-cell *S*. Typhi and *S*. Typhi flagella also have the ability to downregulate *in vitro* lymphocyte proliferation to soluble antigens and mitogens by affecting macrophage function, suggesting that *S*. Typhi components have the potential to exert both up-regulatory and down-regulatory effects on the host immune response (53). Taken together, these observations suggest that although antibodies are likely to participate in protection against typhoid fever, CMI probably represent the dominant protective immune responses that eventually lead to the elimination of these bacteria from the host.

More limited information is available regarding immunological responses in paratyphoid fever. Several reports showed the presence of serological responses against LPS and H-flagellar *S*. Paratyphi antigens using the Widal, colorimetric, and ELISA tests (7, 54). More recently, immunogenic *S*. Paratyphi A proteins expressed in bacteremic *S*. Paratyphi A-infected individuals have been identified using an immunoscreening technique (IVIAT; *in vivo*-induced antigen technology) (4). These studies identified several *S*. Paratyphi A proteins expressed *in vivo* (~20 proteins, including those involved in pathogenesis, such as fimbria, cell envelope and membrane structures, energy metabolism, and cellular proteases), which elicited antibody responses in these patients during the acute and convalescent phases. These results confirmed and extended previous studies by the same group using a different technique (SCOTS, selective capture of transcribed sequences) in Bangladeshi patients who were bacteremic with *S*. Paratyphi A and *S*. Typhi (51, 55). Taken together, these observations highlight several *S*. Paratyphi A proteins, which might play an important role in *S*. Paratyphi A pathogenesis and which may serve as targets of upcoming vaccine development efforts.

Regarding CMI, as reported in typhoid fever, elevated serum levels of IFN-γ, IL-6, TNF-R p55, and TNF-R p75 were reported in *S*. Paratyphi A-infected patients (47). Moreover, a very recent manuscript described the induction of serum pro-inflammatory cytokines in Israeli travelers who became infected with *S*. Paratyphi A while visiting Nepal (6). These studies showed elevated serum levels of both pro-inflammatory and anti-inflammatory cytokines/chemokines during the acute phase, including IFN-γ, IL-6, IL-8, IL-10, IL-15, and TNF-α. Of note, no changes were observed in the serum levels of the other cytokines evaluated in these studies (i.e., IL-1α, IL-1β, IL-2, IL-4, IL-5, IL-12p70, IL-13, IL-17, IL-23, and TNF-β). These increases in pro-inflammatory cytokines/chemokines observed in *S*. Paratyphi A infections are similar to those reported in typhoid fever, supporting the contention that similar host immune responses might be elicited in enteric fevers caused by *S*. Typhi and *S*. Paratyphi bacteria. Interestingly, elevated serum levels of pro-inflammatory (IFN-γ, IL-12, and TNF-α) cytokines but decreased levels of IL-10 were reported in patients with early non-typhoidal gastroenteric *Salmonella* bacterial clearance in stools as compared to the non-clearance group (56). It is reasonable to speculate that these observations demonstrating the increased circulating levels of both pro-inflammatory and anti-inflammatory cytokines/chemokines suggest the concomitant presence of both T effector (T_eff_) and T regulatory (T_reg_) responses following wild-type infection.

Another issue to consider regarding the cytokine/chemokine data in natural infections with typhoidal and non-typhoidal *Salmonella* is that although increases in circulating cytokines/ chemokines are widely considered to be associated with protective responses, this might not necessarily be an accurate interpretation. In fact, it is likely that the levels of cytokines/chemokines in the microenvironments of the gut and the “RES” (e.g., regional lymph nodes, spleen, and other secondary lymphoid tissues) are not necessarily reflected in circulation. These are the sites in which most immune responses are likely to be generated, and where *Salmonella* find their niche(s) for long-term persistence, representing important sites for localized immune responses. With the information currently available, it is not possible to rule out the notion that serum/plasma levels might be a representation of a generalized pro-inflammatory response (part of the so called “cytokine storm,” a surrogate marker of inflammation) in response to a systemic bacterial infection (e.g., the host’s response to LPS and other bacterial antigens) rather than an effective targeted host response leading to protection.

## Immunity Elicited by Experimental Challenge with Wild-Type *S*. Typhi (Controlled Human Infection Model; Typhoid CHI)

*S*. Typhi is a human-restricted pathogen, i.e., there are no good animal models that faithfully recapitulate *S*. Typhi infection (57). To partially address this shortcoming, the infection of susceptible mice with *S*. Typhimurium has been used as a model for the pathogenesis of human typhoid fever (57). Although these murine models have provided considerable knowledge regarding host–pathogen interactions, they do not fully represent *S*. Typhi infection in humans (58). Furthermore, the recent availability of full genome sequences from various *S. enterica* serovars have uncovered many differences in inactivated or disrupted genes, which can explain, at least in part, the dissimilarities observed in the immune and other host responses to these enteric bacteria (58). Thus, controlled human infection (CHI, “challenge”) studies in which subjects are exposed orally to wild-type *S*. Typhi, have the potential to provide a better understanding of the human immune response to infection. Additionally, these studies have the capacity to uncover the correlates of protection against *S*. Typhi, which might prove critical to accelerate the development of better and more effective vaccines to prevent typhoid and other enteric fevers (59, 60).

While challenge experiments with virulent *S*. Typhi were reported early in the twentieth century (59), University of Maryland Researcher, Dr. Theodore E. Woodward, is considered the pioneer in the establishment of a reproducible challenge model (61). In this challenge model, participants were orally challenged with wild-type *S*. Typhi suspended in milk, without buffer. In his first challenge assay performed in the 1950s, Dr. Woodward used the wild-type strain Ty2 isolated from an outbreak in Kherson (in modern day Ukraine) in 1918 (62). All subsequent challenge assays were performed using the Quailes strain, which was isolated from the gallbladder of a chronic carrier, and demonstrated virulence through transmission to several household members (60). To highlight the importance of this challenge model, studies by Dr. Woodward and his collaborators at the University of Maryland led to the successful use of chloramphenicol in the treatment of patients with typhoid fever (61) and also served as the first step toward eventual licensure of the Ty21a typhoid vaccine (63).

Very recently, over three decades after the last human wild-type *S*. Typhi challenge study was performed at University of Maryland, Dr. Pollard’s group in Oxford (UK) has re-established this model. This CHI model followed in the steps of previous studies by challenging healthy adult subjects with wild-type *S*. Typhi Quailes strain (63). However, the challenge agent was suspended in a sodium bicarbonate solution rather than milk. Two dose levels (10^3^ or 10^4^ colony-forming units) resulted in attack rates of 55 or 65%, respectively. Interestingly, participants who developed typhoid infection demonstrated serological responses to flagellin and LPS antigens by day 14, while no changes were observed in the titers of these antibodies in participants not succumbing to infection after challenge. It is reasonable to speculate that the increased anti-LPS responses in subjects who developed typhoid was largely the result of clinical disease involving local and systemic infection rather than representing a protective mechanism at play. Moreover, anti-*S*. Typhi antibody baseline titers did not correlate with subsequent infection risk (63). These results are somewhat different than those from Maryland challenges in which anti-H antibodies appear to correlate with protection. Of note, in the Oxford CHI studies, antibody responses were not detected against Vi, which is present in most *S*. Typhi isolates, including the Quailes strain. These results are in agreement with the Maryland challenge studies, which showed considerable increases in flagellin and LPS antibody titers soon after infection (during the incubation period) but only modest rises in anti-Vi antibody titers (64). Of note, clinical illness and relapse were reported in the Maryland challenge studies to occur at the peak of antibody responses (64). Taken in concert, these results suggest that anti-Vi and other anti-*S*. Typhi-specific antibodies are likely to play a role in protection during natural infection. However, their precise contribution to host defense, either independently or in conjunction with other effector immune responses, remains to be established.

The Maryland CHI studies conducted in the 1950s, 1960s, and 1970s did not address the role of CMI in protection against *S*. Typhi infection, primarily due to the lack of appropriate assays. It is likely, however, that the performance of in depth CMI studies with specimens obtained from subjects participating in the recently re-established Oxford typhoid CHI model using the most advanced current techniques and instrumentation, will greatly advance our understanding of the role of CMI in protection.

## Typhoid and Paratyphoid Vaccines: Current Status

The first typhoid vaccines consisting of inactivated (heat-killed, phenol-preserved) *S*. Typhi delivered parenterally were developed as far back as 1896 by Pfeiffer and Kolle in Germany and Wright in England (65). At that time, typhoid fever was a much-feared disease. However, following the discovery that antibiotics such as chloramphenicol could successfully treat typhoid fever, the interest in typhoid vaccines waned. A resurgence of interest in typhoid vaccines began in the 1970s, when epidemics of chloramphenicol-resistant typhoid occurred in Mexico and Vietnam (1). Although inactivated whole-cell vaccines are immunogenic and effective, due to excessive reactogenicity, they are no longer manufactured (66–68). Currently, there are two vaccines against *S*. Typhi that are licensed in the USA for use in humans, the purified Vi (“virulence”) polysaccharide parenteral vaccine and the oral live-attenuated *S*. Typhi strain Ty21a vaccine. Both vaccines are moderately protective and have been shown to induce herd immunity (69, 70). The Vi polysaccharide vaccine was developed by Robbins and collaborators at NIH as an injectable subunit vaccine and is currently sold by several companies, including Sanofi Pasteur and GlaxoSmithKline (Table 1) (69, 71–75). Although the Vi vaccine confers a moderate level (55–72%) of protection in children over 2 years of age after a single dose, this vaccine does not confer “memory” and there are no robust data to suggest that the efficacy of Vi persists beyond 3 years (66, 67, 69, 76). The Ty21a vaccine, licensed for children older than 6 years, confers a moderate level of long-lived protection (60–80%, 5–7 years) but requires the administration of three to four spaced doses (66, 70, 77). Despite its moderate immunogenicity much of our knowledge regarding immunological responses against *S*. Typhi has been derived from studies of Ty21a immunization (Table 1) (52, 66, 67, 78–92). Vaccination of children younger than 2 years old, however, requires a new approach. The Vi-protein-conjugate vaccines appear promising in this regard (14, 93–96). Conjugate Vi vaccines consist of the *S*. Typhi Vi polysaccharide, a T-cell-independent antigen, covalently bound to a carrier protein. Hence, the conjugation process increases the immunogenicity of the vaccine by converting the Vi polysaccharide into a “T-cell-dependent” antigen. Various Vi-conjugate vaccine candidates are in development. For example, Vi *O*-Acetyl Pectin-rEPA conjugate vaccine, a modified conjugate vaccine where Vi is conjugated to non-toxic recombinant *Pseudomonas aeruginosa* exotoxin A (rEPA) has shown an efficacy of ~90% in 2–5-year-old children (94, 96–99). Recently, Bharat Biotech in India has launched the world’s first Vi-conjugate vaccine, called Typbar-TCV™, consisting of Vi from *S*. Typhi strain Ty2 conjugated to tetanus toxoid (TT) as a carrier protein, which can be given to infants older than 6 months (100, 101). Other vaccine candidates include Vi-conjugated to CRM_197_ (95) and diphtheria toxoid (102) (Table 1). Of note, issues that have been raised and merit consideration regarding the use of Vi and Vi-conjugate vaccines are the emergence of *S*. Typhi Vi antigen-negative strains in multidrug-resistant typhoid fever cases and the possibility that the generalized use of Vi vaccines might lead to increased incidence of enteric fevers caused by Vi-negative strains for which Vi vaccines will be ineffective (103, 104). As a result of these issues, as well as other scientific, logistical, and economic reasons, additional subunit vaccine candidates are being actively developed for the prevention of enteric fevers. These include, among others, conjugates of *S*. Typhi and *S*. Paratyphi A LPS to carrier proteins or *Salmonella* proteins (e.g., flagellin, porins) to extend the generation of immunity to other relevant specific antigens (101).

**Table 1:** **Selected licensed *S*. Typhi vaccines and vaccine candidates**.

Type of vaccine	Trade name	Licensed	Manufacturer/ developer	Number of doses	Efficacy (field trials)	Minimum age for administration	Immunogenicity data	Reference
Inactivated whole cell	N/A	Yes	No longer being manufactured	2	~60–80%	N/A	Serum antibodies, lymphocyte proliferation, PBMC migration inhibition	(66 –68 )
Live attenuated	Ty21a (Vivotif ^®^)	Yes	Crucell Switzerland Ltd	3–4	~60–80%	≥6 years	Serum antibodies, ASC, ALS, ADCC, opsonophagocytosis, B memory, lymphocyte proliferation, production of multiple cytokines, and chemokines, CTL activity, cross-reactivity with *S*. Paratyphi A & B	(52, 66, 67, 78–92)
	CVD 906	No	CVD–UMB	1	N/A	N/A	Serum antibodies, jejunal IgA, ASC, lymphocyte proliferation, IFN-γ and IL-6 production	(105–107)
	CVD 908	No	CVD–UMB	1	N/A	N/A	IgA ASC, serum IgG, lymphocyte proliferation, IFN-γ and IL-6 production	(107–109)
	CVD 906-htrA	No	CVD–UMB	1	N/A	N/A	Serum antibodies, jejunal IgA, ASC, lymphocyte proliferation	(110 )
	CVD 908-htrA	No	CVD–UMB	1	N/A	N/A	Serum antibodies, jejunal IgA, ASC, lymphocyte proliferation, IFN-γ production	(110, 111)
	CVD 909	No	CVD–UMB	1	N/A	N/A	Serum antibodies, ASC, ALS, B memory, opsonophagocytosis, lymphocyte proliferation, cross-reactivity against *S*. Paratyphi A and B	(89, 90, 112 )
	Ty800	No	Massachusetts General Hospital	1	N/A	N/A	IgA ASC, serum IgG and IgA	(113 )
	M01ZH09	No	Microscience Limited	1	N/A	N/A	Serum antibodies, ASC, ALS, opsonophagocytosis, bactericidal, lymphocyte proliferation, IFN-γ production	(114–118)
	χ3927	No	CVD–UMB	1	N/A	N/A	Serum antibodies, Jejunal sIgA, ASC	(105 )
**SUBUNIT**								
Vi polysaccharide	Typhim Vi^®^	Yes	Sanofi Pasteur	1	55–72%	≥2 years	Serum antibodies	(66, 67, 72)
	Typherix ^®^	Yes	GlaxoSmithKline	1	61%	≥2 years	Serum antibodies	(66, 67, 69)
	Typbar^®^	Yes	Bharat Biotech	1	N/A	≥2 years	Serum antibodies	(73)
	Vax-TyVi^®^	Yes	Finlay Instituto	1	N/A	≥5 years	Serum antibodies	(74)
	TyViVac	Yes	Dalat Vaccine Company (DAVAC)	1	N/A	≥2 years	Serum antibodies	Product insert
	BioTyph^TM^	Yes	BioMed	1	N/A	≥2 years	Serum antibodies	Product insert
Vi combination	Hepatyrix^TM^ (hepatitis A and Vi)	Yes	GlaxoSmithKline	1	N/A	≥15 years	Serum antibodies	(75)
	ViATIM^®^ (hepatitis Aand Vi)	Yes	Sanofi Pasteur	1	N/A	≥16 years	Serum antibodies	(75)
Vi conjugate	Pedatyph^TM^ (Vi-TT)	Yes	BioMed	2	N/A	≥3 months	Serum antibodies	(93)
	Typbar-TCV^TM^ (Vi-TT)	Yes	Bharat Biotech	1	N/A	≥6 months	Serum antibodies	(100, 101)
	Vi-rEPA	No	NIH	2	89%	≥2 years	Serum antibodies	(94, 96, 99)
	Vi-CRM_197_	No	Novartis Vaccines Institute	1	N/A	18–40 years	Serum antibodies	(95 )

*N/A, not available; CVD–UMB, Center for Vaccine Development–University of Maryland Baltimore; PBMC, peripheral blood mononuclear cells; ASC, antibody secreting cells; ADCC, antibody dependent cellular cytotoxicity; CTL, cytotoxic T lymphocyte; ALS, antibodies in lymphocyte supernatant; IFN-γ, interferon-γ; (IL-6) interleukin-6, NIH, National Institutes of Health; TT, tetanus toxoid; rEPA, non-toxic recombinant *Pseudomonas aeruginosa* exotoxin A; CRM_197_, mutant diphtheria toxin carrier protein*.

Because of the above considerations, investigators, including those at the University of Maryland Center for Vaccine Development (CVD), have engineered new attenuated typhoid vaccine strains that aim to be as safe as Ty21a but immunogenic and protective following the ingestion of only a single dose. These vaccine candidates include Ty800 (113), M01ZH09 (114–120), and others based on attenuation of *S*. Typhi by deletions of genes such as those involved in the synthesis of aromatic amino acids (*aroC*, *aroD*) and heat-shock proteins (*htrA*). The latter vaccine candidates, designated CVD 906 (105, 106), CVD 908 (107–109), CVD 908-*htrA* (110), and CVD 909 (112), have been evaluated in volunteers and shown to induce potent CMI both *in vitro* and *ex vivo* (83–85, 105, 107, 110–112, 121–123), as well as humoral responses (105, 108, 110, 112) (see below for details). Except for CVD 906, these strains are derived from the wild-type *S*. Typhi Ty2 strain, the same strain from which the Ty21a vaccine was derived. Table 1 includes a summary of the characteristics of these typhoid vaccine strains and the documented immune responses elicited in volunteers.

Regarding *S*. Paratyphi vaccines, the first killed whole-cell parenteral typhoid vaccines produced a century ago consisted of a trivalent combination of heat-inactivated and phenol-preserved *S*. Typhi, *S*. Paratyphi A, and *S*. Paratyphi B (TAB vaccine) (67). Although this vaccine was moderately efficacious, its manufacture was discontinued due to high levels of reactogenicity (2). Although several vaccine candidates against enteric fever caused by *S*. Paratyphi A are at various stages of development, including *S*. Paratyphi A O-specific polysaccharide-TT and CRM_197_ conjugates (124–126), no vaccines are currently commercially available.

It is important to note that there has been considerable interest in exploring the use of attenuated *S*. Typhi strains as live-vector vaccines. *S*. Typhi presents multiple advantages as a live-vector, including (a) oral delivery, (b) targeting of M cells overlying gut-associated lymphoid tissue (inductive sites for immune responses), (c) internalization by DC and macrophages, and (d) stimulation of broad immune responses (127). Indeed, multiple clinical trials have been performed to investigate the immunogenicity of genetically engineered *S*. Typhi expressing foreign antigens (111, 127–134). While these studies have detected only modest immune responses against the foreign antigens, novel engineering strategies hold great potential to enhance the immunogenicity of such vaccines (127). This remains an important avenue of research and improved understanding of immune responses elicited by *S*. Typhi and *S*. Paratyphi A vaccines may facilitate these efforts.

## Adaptive Responses to *S*. Typhi in Volunteers Immunized with Licensed Typhoid Vaccines and Vaccine Candidates

**Figure 1:**
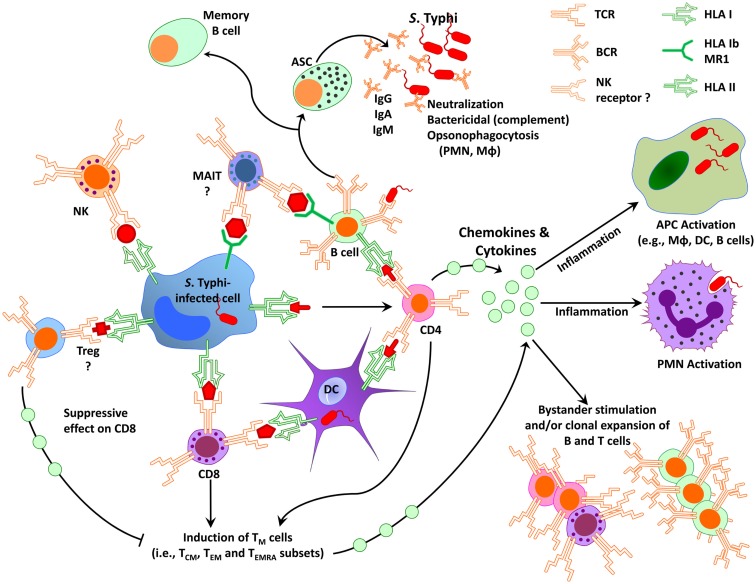
**Simplified diagram of immunity to *S*. Typhi in humans**. Immunity to *S*. Typhi is extremely complex involving multiple antigen-presenting cells (e.g., macrophages, dendritic cells, B cells) and effector cells (e.g., various effector and regulatory T-cell subsets, B cells, NK, and MAIT cells). APC, antigen-presenting cells; ASC, antibody secreting cells; DC, dendritic cells; CD8, CD8^+^ T-cells; CD4, CD4^+^ T-cells; MAIT, mucosal associated invariant T-cells; Mφ, macrophages; NK, natural killer cells; PMN, polymorphonuclear neutrophil; T_M_, memory T-cells; T_CM_, central memory T-cells; T_EM_, effector memory T-cells; T_EMRA_, effector memory expressing CD45RA; T_reg_, regulatory T-cells; HLA, human leukocytes antigen; HLA-I, HLA class I; HLA-II, HLA class II; BCR, B cell receptor; TCR, T-cell receptor; MR1, HLA-I non-classical (b) molecule MR1; Ig, immunoglobulin.

As discussed above, immunity to *S*. Typhi is complex involving antibodies and CMI (135–138). Because *S*. Typhi is a facultative intracellular bacterium, we and others have hypothesized that both antibodies and CMI might play complementary roles in protection from infection. While antibodies are likely to play an important role in defense against extracellular bacteria, CMI is expected to be essential in eliminating *S*. Typhi-infected cells. Based on results from studies using specimens from subjects immunized with attenuated typhoid vaccines, we surmise that serum antibodies, SIgA, CD4^+^, CD8^+^, and other T-cell subsets (e.g., mucosal associated invariant T-cells, MAIT), as well as the interaction between T, B, and antigen-presenting cells (APC, e.g., macrophages, DC) are all likely to contribute to an effective acquired immune response against typhoid fever (Figure 1). However, the relative contribution of each main arm of the effector immune response, i.e., humoral and cellular, and the antigen specificity of the responses remain largely unknown. Below, we will critically address the key humoral and CMI responses, which we believe are essential in generating “protective” immunity against *S*. Typhi infection, as well as discuss current gaps in knowledge, which need to be addressed to enable the identification of immunological correlates of protection in enteric fevers.

### Humoral responses

#### Antibodies

Numerous studies have reported serum antibody production following *S*. Typhi infection and immunization. Antibodies against the O antigen of *S*. Typhi LPS, the Vi antigen, and the H antigen are routinely measured as markers of immunogenicity following *S*. Typhi immunization (67, 110, 112–114, 139, 140). Despite extensive study, the precise role that antibodies play in protection against *S*. Typhi remains unknown. As discussed above, relapses of typhoid fever occur in individuals despite elevated titers of serum anti-*S*. Typhi antibodies (34, 141) and in a recent human challenge with wild-type *S*. Typhi, pre-challenge levels of anti-H and Vi antibodies did not correlate with protection (63). These studies showed that volunteers who were diagnosed with typhoid demonstrated increases in IgG, IgM, and IgA to LPS and H antigens while little change was seen in volunteers who did not succumb to the disease (63). Anti-Vi levels remained unchanged throughout the study (63). Nevertheless, the fact that Vi polysaccharide vaccines can induce protection against typhoid indicates that high-anti-Vi antibodies are protective. In fact, defined levels of serum anti-Vi antibodies (1.4–2.0 μg/ml) have been reported to act as a serological surrogate of protection in Vi-rEPA conjugate vaccine efficacy trials (94). Presumably, anti-Vi antibodies function by counteracting the evasion of innate immune recognition in the intestinal mucosa and obstruction of bacterial-guided neutrophil chemotaxis, which have been proposed as possible mechanisms by which Vi subverts host immune responses (142, 143). Interestingly, the live-attenuated oral vaccine Ty21a, which lacks the Vi antigen, results in similar levels of protection as those of the Vi polysaccharide vaccine (12), indicating that multiple adaptive immunological responses can lead to effective protection (Table 1). In field studies of an enteric-coated capsule formulation of Ty21a, seroconversion, as measured by anti-O IgG, correlated with protection (67, 144). However, in these same clinical trials the seroconversion rate of IgG O antibodies did not predict the poor efficacy of other vaccine formulations (67). Seroconversion against *S*. Typhi-O antigen has, nevertheless, been used as a marker of immunogenicity following immunization with single-dose live-attenuated vaccine candidates (110, 112–114, 140). In addition to serum antibodies, *S*. Typhi-specific IgA can be found in saliva, intestinal fluids, and stools following oral immunization with live-attenuated *S*. Typhi or natural infection (26, 78, 145, 146).

Immunoglobulins can be divided into subclasses (e.g., IgA1 and IgA2) based on structural, antigenic, and functional differences (147). The subclasses of IgA are not evenly distributed among bodily fluids with IgA1 dominating in serum and IgA2 found primarily in secretions. In individuals immunized with Ty21a vaccine, *S*. Typhi-specific IgA1 predominated in serum, saliva, and tears, while IgA2 predominated in intestinal lavage fluid (146). IgG can be subclassified into IgG1-4 with different subclasses typically responding to different types of antigen. For example, IgG1 and IgG3 are generally induced by protein antigens, while IgG2 and IgG4 antibodies are associated with polysaccharide antigens (147). Interestingly, however, serum antibodies against *Salmonella* LPS belong primarily to the IgG1, IgA1, and IgA2 subclasses (148). In contrast, as expected, IgG2 anti-Vi was found to be the predominant IgG subclass in a Vi polysaccharide vaccine study in Nepal (33). Moreover, following a single subcutaneous dose of an *S*. Typhi vaccine candidate containing porins (protein antigen) IgM and both IgG1 and IgG2 seroconversions were detected (136). Unfortunately, no information is available on the avidity of anti-*S*. Typhi antibodies elicited by natural infection or immunization. This is a key measurement of the strength of the attachment of antibodies to their antigen, which is highest after B cells have been adequately primed and is an important measurement of the strength of the anamnestic response. Further understanding of the specific immunoglobulin subclasses and avidity associated with protective responses will be of importance in informing decisions regarding vaccine development.

Despite the large amounts of data regarding production of antibodies against *S*. Typhi, there have been few investigations of the functional properties of these antibodies. Early studies indicated that *S*. Typhi-specific IgA was responsible for antibody dependent cellular cytotoxicity (ADCC) following Ty21a immunization (87). In Nepal, an *S*. Typhi endemic region, bactericidal activity of serum was shown to increase with age; however, no correlation was found between bactericidal titer and anti-Vi titers (33). Recently, we, and others, have reported the induction of functional opsonophagocytic bactericidal *S*. Typhi-specific antibodies that might assist in the elimination of *S*. Typhi (90, 118). These opsonophagocytic antibodies appear to be of the IgG isotype. Further investigation of these functional antibodies may lead to improved measures of immunogenicity and might prove to be more closely associated with protective immunity than antibody measurements by ELISA.

In sum, the sometimes conflicting and fragmentary data regarding the role of antibodies in defense against *S*. Typhi suggest that while they may contribute to an effective response, they are unlikely to represent the dominant mediator of protection in humans following exposure to wild-type organisms.

#### B cells

Although, studies in knockout mice indicate that B cells play an important role in protection against *S*. Typhimurium (149), the precise role that B cells play in protection against *S*. Typhi in humans remains unknown. Antibody production is clearly a major function of B cells; however, B cells also contribute to immune responses via antigen presentation, cytokine production, and the initiation of T-cell responses. For example, *Salmonella*-specific primary human B cells are able to internalize *S*. Typhimurium via their B cell receptor and stimulate a strong recall response by cytotoxic CD8^+^ T-cells (150). In fact, following internalization, *Salmonella* survive in the B cell and antigens are loaded onto MHC class I for cross-presentation to CD8^+^ T-cells (150). These results are supported by our previous observations showing that *S*. Typhi-infected B cells can serve as excellent APC for *S*. Typhi antigens. We reported that Epstein–Barr virus (EBV)-transformed lymphoblastoid B cell lines (B-LCL) are able to effectively stimulate CD4^+^ cells as well as classical and non-classical CD8^+^ cells (82–85, 92, 121–123, 151, 152). These findings also re-emphasize the importance of communication among immune cell compartments and the possibility that B cells contribute to host defense from *S*. Typhi infection through mechanisms beyond their primary role in antibody production (20) (Figure 1).

##### Antibody secreting cells

A key aspect of B cells is their ability to undergo cell differentiation and become antibody secreting cells (ASC) (153). In *Salmonella* infection, specific ASC circulate briefly systemically, peaking at ~7–10 days after antigen encounter, before homing to mucosal effector sites (91, 110, 112, 113, 115, 140, 144, 154–157). However, prolonged exposure to antigen results in extended circulation of *S*. Typhi-specific ASC in peripheral blood (158). In fact, patients with prolonged diarrhea have circulating ASC throughout the duration of the pathogen exposure (158). Following mucosal antigen encounter (i.e., oral immunization), *S*. Typhi-specific IgA ASC predominate followed by substantial IgM ASC and low numbers of IgG ASC (79, 158). Of note, the magnitude of ASC response displays considerable inter-individual variation. Three main factors appear to dictate the magnitude of the response: antigen type (live versus killed), number of vaccine doses ingested, and formulation of the vaccine (158). Specifically, immunization with a live oral vaccine resulted in higher magnitude of ASC responses compared to a killed vaccine (159). Ingestion of three doses of vaccine resulted in higher numbers of *S*. Typhi-specific ASC than did two doses and, although there was no further increase in the peak number of ASC following six doses, the response remained higher for a longer duration (158). Additionally, different vaccine formulations (i.e., gelatin capsules, enteric-coated capsules, suspension) showed different magnitudes of response, with the suspension-formulation, resulting in the highest number of *S*. Typhi-specific ASC (79, 158). Notably, the magnitude of the IgA ASC response against the O antigen induced by different formulations and schedules of Ty21a correlated with the efficacy shown in field trials of the same formulations and schedules (79, 144). Other studies showed that the serum antibody increased concomitantly with increasing ASC numbers, and that, when ASC numbers were low, serum antibody responses were undetectable (79). Consequently, it has been proposed that detection of ASC is a more sensitive measurement of immunogenicity than serum antibody titers. The homing patterns of *S*. Typhi-specific ASC have been rigorously studied and are discussed in detail below.

##### Memory B cells

It is widely accepted that immunological memory is of critical importance for the development of long-lasting protective responses following immunization (160). Memory B cells (B_M_) are long-lived antigen primed cells that upon antigenic stimulation during a secondary response undergo rapid terminal differentiation into plasmablasts and plasma cells (161). While there are multiple classification methods to define this heterogeneous population, most B_M_ are widely accepted to exhibit the phenotype CD19^+^ CD27^+^ IgD^+/−^, although a minor B_M_ subset lacking CD27 expression has also been reported (162). Of note, it has been reported that B_M_ are able to mature either inside or outside of the germinal centers and that this phenomenon may be T-cell-dependent or independent (161, 163). We have recently made the novel observation that immunization with attenuated *S*. Typhi vaccines elicits CD19^+^ CD27^+^ B_M_ specific for *S*. Typhi antigens (e.g., LPS, flagella, Vi) (89) and described the longevity (up to 1 year), magnitude, and characteristics of these responses (89). Notably, strong B_M_ responses against both T-cell-dependent (flagella) and T-cell-independent (LPS and Vi) antigens were identified in volunteers primed with CVD 909 (a Vi expressing live-attenuated *S*. Typhi vaccine candidate). These results suggest that immunization with CVD 909 was capable of mucosally priming the immune system to deliver robust and sustained Vi-specific B_M_ responses to a subsequent parenteral exposure. LPS-specific B_M_ responses were also observed in volunteers primed with CVD 909, but these responses were of lower magnitude than those against Vi. Similar to findings for ASC, LPS-specific IgA B_M_ cells predominated over LPS-specific IgG B_M_ responses. In the same study, we observed that volunteers immunized with Ty21a also developed IgA B_M_ responses to LPS, but only a single volunteer developed IgG B_M_ responses against LPS. Moreover, both CVD 909 and Ty21a were capable of inducing anti-*S*. Typhi flagella IgG and IgA B_M_ responses. Finally, we observed a strong association between the frequency of antigen-specific B_M_ cells and antibody levels, supporting an important role of this cell population in the generation of humoral responses. Recent studies have shown that *S*. Typhi porins can induce short- and long-lasting IgG and IgM responses in humans, a response likely to be mediated by B_M_ (136). Interestingly, studies in mice have also identified IgM B_M_, which are likely to secure long-term production of bactericidal IgM antibodies following inoculation with *S*. Typhi porins (164). This study also reported the induction of Type 1 T follicular helper (Tfh) cells that produce IFN-γ, which are thought to support the generation of these B_M_ (164). However, the relative contribution of the various B_M_ and Tfh subsets to enduring protection remains to be determined. Further characterization of these responses and cell subsets may help elucidate mechanisms of sustained protection against *S*. Typhi.

##### B cell phosphorylation

Early signaling events that occur following encounter of B cells with *S*. Typhi and other pathogens are of critical importance in the generation of cellular responses. Recently, we described the phosphorylation patterns associated with *S*. Typhi-specific B cells (165). We reported that exposure of PBMC from healthy volunteers to fluorescently labeled, heat-killed *S*. Typhi resulted in bacterial binding to naïve and unswitched memory (Um) B cells as detected by flow cytometry. Although naïve B cells that interacted with *S*. Typhi were observed, phosphorylation of Stk, Akt, and p38MAPK were not identified in this subset. In contrast, Um B cells showed multi-phosphorylation of all three proteins assayed, as well as cells that phosphorylated only p38MAPK or Akt and p38MAPK. Interestingly, different antigenic structures appeared to induce different patterns of phosphorylation. For example, the phosphorylation patterns induced by *S*. Typhi were dramatically different from the phosphorylation patterns induced by the Gram-positive bacterium *Streptococcus pneumoniae*. These novel studies provide the first glimpse of the activation pathways of *S*. Typhi-specific B cell responses in humans. Further characterization of these mechanisms can provide key information to help advance the generation of novel vaccine strategies.

##### B cell homing

Although most of our knowledge of immune responses against *Salmonella* in humans is derived from studies using peripheral blood, effector immunity in the local microenvironment of the gut is likely to be of paramount importance in the understanding of protection against *S*. Typhi infection. Mucosal derived circulating IgA ASC detected after administration of live oral typhoid vaccines have been used to estimate the degree of priming of the local intestinal immune system (137). These cells are believed to home to the lamina propria of the intestinal mucosa where they will synthesize and release antibodies (166). Selective homing of cells (including plasmablasts) to the small intestine is believed to be largely driven by the expression of integrin α_4_β_7_ and chemokine (C–C motif) receptor (CCR)9 (167), while CCR10 expression appears to be involved in homing to “common” mucosal tissues (168). The primary site of antigen encounter has been shown to affect the expression of homing receptors on ASC (169). Following mucosal antigen delivery by Ty21a administration, robust migration of *S*. Typhi-specific IgM and IgA ASC toward chemokine (C–C motif) ligand (CCL)25 and CCL28, the ligands for CCR9 and CCR10, respectively, were noted (170). In contrast, systemically derived tetanus-specific ASC did not migrate toward either CCL25 or CCL28, supporting the mucosal specificity of these ligands. Previous work has shown that after oral antigen administration, the majority of ASC produce the mucosal Ig-isotype, IgA, and all of them express the gut homing receptor, integrin α_4_β_7_, thus, implying mucosal homing of these cells (137, 154, 157, 158). Moreover, when comparing oral Ty21a and parenteral Vi-conjugate vaccines, Ty21a but not Vi immunization recapitulates the homing receptor profile of ASC occurring in natural infection (e.g., integrin α_4_β_7_ expression) (155). We have recently shown that sorted IgG and IgA ASC recognizing *S*. Typhi-LPS are predominantly CD19^+^ CD27^+^ (a phenotype associated with B_M_ and plasmablasts) with selective gut homing potential (e.g., integrin α_4_β_7_^+^ CD62L^−^) (91). Of note, however, both IgG and IgA cells were also observed among integrin α_4_β_7_^+^ CD62L^+^, suggesting that they have the capacity to home to the gut, as well as peripheral lymph nodes, and perhaps other secondary lymphoid tissues. Further studies of the homing potential of *S*. Typhi-specific B_M_ and plasmablasts is of critical importance to further our understanding of the mechanisms underlying the induction of antigen-specific cells which have the ability to home to the gut (the initial site of infection), as well as to other lymphoid tissues where *S*. Typhi resides following systemic dissemination.

### Cell mediated immune responses

As for other intracellular infections, CMI responses against *S*. Typhi infection rely largely on two types of cells: CD4^+^ and CD8^+^ T-cells (51, 81, 138, 171). The presence of both CD4^+^ helper T-cells and classical class Ia and non-classical HLA-E-restricted *S*. Typhi-specific CD8^+^ T-cells have been observed in individuals with typhoid fever or immunized with Ty21a and other attenuated leading typhoid vaccine candidates, including CVD 908-*htrA* and CVD 909 (51, 82–85, 88, 92, 114, 122, 123, 152, 172). A succinct description of these responses follows.

#### T-cell responses

We, and others, have reported that *S*. Typhi can stimulate the production of an array of pro-inflammatory cytokines including IFN-γ by specific CD4^+^ and CD8^+^ T-cells following immunization (52, 84, 85, 88, 107, 114, 121). For example, IFN-γ production by CD4^+^ and CD8^+^ T-cells in response to *S*. Typhi LPS and flagella antigens has been shown up to 56 days after immunization with attenuated *S*. Typhi vaccines (84, 107, 121). Similarly, in subjects immunized with Ty21a, it has been shown that *S*. Typhi GroEL triggers IFN-γ production by CD8^+^ cells (85). In addition, *S*. Typhi immunization elicits the generation of cytotoxic CD8^+^ T-cells (84, 121, 122). Cytotoxic CD8^+^ T-cells induce apoptosis within minutes of contact with their target by at least two lytic mechanisms (173–175). One, based on granular exocytosis involving perforin and granzymes (176), and another involving a molecule called FAS or APO-1 (177). Using PBMC from individuals immunized with the Ty21a typhoid vaccine (85) and the vaccine candidate strain CVD 909 (123), we have shown that the killing of *S*. Typhi-infected targets by specific CD8^+^ T-cells is largely through a FAS-independent, granule-dependent pathway. These findings were confirmed using two types of autologous target cells: phytohemagglutinin (PHA)-stimulated PBMC, as well as B-LCL (85, 123). Interestingly, killing of these targets involved antigenic presentation by both classical class Ia and non-classical HLA-E molecules indicating that multiple mechanisms might be involved in killing of *S*. Typhi-infected cells (84, 85, 121).

Cell mediated immunity against *S*. Typhi mediated by CD4^+^ and CD8^+^ T-cells appears to depend on the nature of the stimulant. CD4^+^ cells were more prone to respond to *S*. Typhi soluble antigens while CD8^+^ cells were more likely to be activated by *S*. Typhi-infected targets (84, 121, 138, 152). These results emphasize the importance of selecting the appropriate type of stimulant when designing experiments aimed at evaluating T-cell responses. Another important issue related to the host’s response to *S*. Typhi is the dichotomy between T-cell and humoral responses observed in individual subjects. In the past, our group and others have tried exhaustively, and failed, to observe a correlation on a volunteer by volunteer basis between serum antibody titers to *S*. Typhi LPS and/or *S*. Typhi flagella and CMI in individuals immunized with various attenuated *S*. Typhi vaccine strains (42, 107, 121, 140). These observations support the contention that the development and dominance of humoral and/or CMI responses in individual volunteers is likely multifactorial and influenced by individual host factors (e.g., genetic makeup, gut microbiome composition).

On the basis of the expression of defined surface molecules, T-cells can be simplistically subdivided into two main subsets: naïve and memory T (T_M_) cells. Induction of strong and persistent memory T-cell responses is one of the hallmarks of successful vaccination (160, 171). Although T_M_ can be divided into a multitude of subsets, it is widely accepted that the main T_M_ subsets are central memory T-cells (T_CM_), and effector memory T-cells (T_EM_) (178, 179). T_CM_ express surface molecules for memory (e.g., CD45RO), as well as the chemokine receptor CCR7 and CD62L (L-selectin) molecules, which allow efficient homing to peripheral lymph nodes (178, 179). T_EM_ also express CD45RO, but down-regulate the expression of CCR7 and CD62L, which allows them to circulate and migrate to the spleen and non-lymphoid tissues. In humans, some CD8^+^ T_EM_ lack the expression of CD45RO and express CD45RA, a molecule present on naïve T-cells. This subset is termed T_EMRA_ or “terminal memory” cells (178, 179). Recently, we provided the first demonstration of the induction and longevity (up to 2 years) of T_CM_, T_EM_, and T_EMRA_ multifunctional HLA-E restricted CD8^+^ T_M_ cells after Ty21a immunization, suggesting that these cells are important in long-term immunity to *S*. Typhi (82). In these experiments, we showed that following Ty21a vaccination, multiple pro-inflammatory cytokines/chemokines (including IFN-γ) are produced by CD8^+^ T-cells in response to stimulation with *S*. Typhi-infected targets, and that these responses are multiphasic in nature (82). We also observed a striking correlation among subjects who showed strong CD8^+^ T_CM_ subsets and produced IL-2 and IFN-γ at early times and the presence of long-term immune responses (82). We speculated that this phenomenon might be due to the fact that IL-2 and/or IFN-γ-secreting CD8^+^ T_CM_ subsets at early times after vaccination result in the development of a larger pool of long-lived specific CD8^+^ T_M_ cell subsets (e.g., CD8^+^ T_CM_, T_EM_ and T_EMRA_ subsets), which could lead to improved control against re-infection. Recently, these results were confirmed and extended using multichromatic flow cytometry to measure six cytokines simultaneously (IL-10, IL17A, IL-2, IFN-γ, TNF-α, and MIP-1β) (92). In this work, our group demonstrated, for the first time, the presence of IL-17A-producing CD8^+^ cells in Ty21a vaccinees (92). These findings are of great significance since consensus is emerging that multifunctional CD4^+^ and CD8^+^ T-cells are important in determining the effectiveness of immunity to either vaccination (180) or exposure to intracellular microorganisms in humans, including HIV (181, 182) and *Mycobacterium tuberculosis* (183, 184).

It is important to highlight that the balance between suppressive and pro-inflammatory responses might be of critical importance in the host’s ability to mount effective immune responses. For example, experiments in mice have shown that the equilibrium between suppressive T_reg_ and pro-inflammatory T_eff_ responses influence the clearance or persistence of *S*. Typhimurium (185). T_reg_ are characterized by the expression of high levels of the IL-2 receptor (CD25) and transcription factor Forkhead box P3 (FoxP3). Activated T_reg_ may traffic to the sites of specific immune responses and exert their regulatory functions via cell–cell interactions [i.e., cytotoxic T lymphocyte antigen-4 (CTLA-4) competition for co-stimulatory molecules (CD80 and CD86) on APC], consumption of IL-2, and production of anti-inflammatory factors [i.e., IL-10 and transforming growth factor (TGF)-β] (186). Observations in humans, including IL-10 production by PBMC from volunteers immunized with Ty21a and CVD909 in response to *S*. Typhi flagellar antigen (52, 123) and IL-10 detection in the sera of individuals during *S*. Paratyphi A infection (6) indicate a potential role for T_reg_ in establishing a balanced immune response against *S*. Typhi and *S*. Paratyphi infections. Despite these intriguing observations, the role of T_reg_ following *S*. Typhi or *S*. Paratyphi infection or immunization in humans remains unknown.

#### Background T-cell responses and their possible role in controlling *Salmonella* infection

A common finding when measuring T-cell immune responses in humans vaccinated against enteric bacteria, such as *S*. Typhi, is the presence of background *S*. Typhi-specific responses among individuals prior to immunization, even in the absence of travel to endemic areas (81, 82, 84, 92, 121, 123, 136, 152). These background responses are characterized by the presence of specific immune responses against antigens from enteric bacteria in individuals with no history of immunization against, or infection with, the enteric pathogen. Although this background is rather variable, with higher levels observed in individuals in regions with limited sanitation systems (unpublished observations), this phenomenon has been observed in subjects across the World. A prevailing hypothesis is that these background responses are due to the presence of cross-reactive T-cells acquired during previous infections by other enteric pathogens (81, 136, 151) or reacting to the normal gut microbiota (187–190). Although it is difficult to contest these possibilities, it is reasonable to hypothesize that defined subset(s) of T-cells such as innate-like T-cells, including TCRγδ T-cells, NK-T-cells, and MAIT, are responsible, at least in part, for the observed background responses (151). For example, TCRγδ T-cells and NK-T-cells from healthy volunteers with serum antibodies against non-typhoidal *Salmonella* have been reported to produce higher amounts of IFN-γ as compared to conventional CD4^+^ and CD8^+^ T-cells in response to stimulation with *Salmonella* antigens (191). It is also known that MAIT cells play an important physiological role in host bacterial defense and may also be involved in inflammatory disorders, particularly at mucosal surfaces (192–194). Previous work has demonstrated that MAIT cells may play a significant role in *M. tuberculosis* and HIV infections in humans. Gold and colleagues have shown in humans that MAIT cells are decreased in the blood of patients with active TB infection. Other reports have shown that the levels of MAIT cells were severely reduced in circulation in patients with HIV-1 infection (195, 196). Their decline was associated with the time of diagnosis (196) and may reflect diverse mechanisms including their accumulation in tissues and activation and functional exhaustion (195, 196). Of note, a recent study from our group has shown that MAIT cells can be activated by B cells infected with various bacteria (commensals and pathogens from the Enterobacteriaceae family, including *S*. Typhi), but not by uninfected cells (151). These responses were restricted by the non-classical MHC-related molecule 1 (MR1) and involved the endocytic pathway. Moreover, the quality of these responses (i.e., cytokine profiles) were dependent on bacterial load but not on the level of expression of MR1 or bacterial antigen on B cell surface (151). Based on these studies, it is reasonable to speculate that baseline responses by functionally active innate-like T-cells (e.g., TCRγδ T, NK-T, MAIT) and/or those elicited early upon microbial stimulation by vaccination or acute infection, might contribute to prevent *S*. Typhi infection. These cell subsets may be responsible for controlling the infection soon after exposure (subclinical infection), and contributing to clear the infection without causing overt disease once the specific adaptive immune responses are fully developed.

#### Dendritic cell cross-presentation and CD8^+^ T-cells

The mechanism(s) underlying *S*. Typhi regulation of the development of specific T-cell responses in humans remains unclear. Studies in mice have shown that DC can either directly (upon uptake and processing of *Salmonella*) or indirectly (by bystander mechanisms) elicit *Salmonella*-specific CD8^+^ T-cells (197). DC are APC that have a strategic function in the initiation and modulation of the immune responses (198). In addition to presenting exogenous antigens using the conventional MHC class II activation pathway typically used by CD4^+^ T-cells, these cells have developed an alternative pathway where exogenous antigens can be presented through an MHC class I activation pathway to CD8^+^ T-cells (198). This alternative pathway is called the cross-presentation pathway (199). Although multiple APC are able to cross-present antigens, DC are the most efficient *in vivo* (200). Therefore, the successful generation of strong CD8^+^ T-cell responses to vaccine antigens might be linked to the modulation of the DC cross-presentation.

Our group has provided the first direct demonstration in humans that DC, through suicide cross-presentation, uptake *S*. Typhi-infected human cells and release IFN-γ and IL-12p70, leading to the subsequent presentation of bacterial antigens and triggering the induction of mostly CD3^+^CD8^+^CD45RA^−^CD62L^−^T_M_ cells (201). We observed that upon infection with live *S*. Typhi, human DC produced high levels of the pro-inflammatory cytokines IL-6, IL-8, and TNF-α but low levels of IL-12 p70 and IFN-γ (201). In contrast, DC co-cultured with *S*. Typhi-infected cells produced high levels of IL-12 p70, IFN-γ, and TNF-α (201). These interesting and novel findings are in agreement with previous work showing that IL-12 and IFN-γ are essential for resistance to *Salmonella* infection in mice (21, 202, 203), and that they are likely to also be important in humans (38, 56). Thus, it is reasonable to speculate that cross-presentation of vaccine antigens to CD8^+^ T-cells might be an important mechanism of antigen presentation leading to the generation of protective immune responses against *S*. Typhi infection.

#### T-cell homing

Migration or “homing” is a multi-step process where the adhesion of lymphocyte surface homing receptors to their counterparts, addresins, on endothelial cells is the key step (204). As with B cells, the selective homing of effector memory cells to the lamina propria of the small intestine is driven, to a large extent, by the expression of integrin α_4_β_7_ and CCR9 (205–209). For example, virtually all T-cells in the small intestine express CCR9 (206). Another molecule implicated in this process is integrin αEβ7 (CD103), which is present in a subset of CCR9^+^ T-cells (210).

Generation of specific memory CD4^+^ and CD8^+^ T-cells with gut homing potential following oral typhoid immunization has been well described (81, 83, 152). Previous work has shown that sorted integrin β7-expressing memory T-cells (CD45RA^−^ β7^high^ cells) from volunteers immunized with *S*. Typhi vaccine strain Ty21a when stimulated *in vitro* produced around 10-fold more IFN-γ than the remaining populations (CD45RA^−^ β7^−^ or CD45RA^−^ β7^intermediate^) (81). Also, using cells from volunteers immunized with the candidate *S*. Typhi vaccine strain CVD 909, our group further characterized the gut homing potential and induction of IFN-γ production in the central (T_CM_, CD45RO^+^ CD62L^+^) and effector (T_EM_, CD45RO^+^ CD62L^−^) memory T populations (152). Interestingly, we observed that the homing potential of CD4^+^ and CD8^+^ T_M_ subsets were distinct. Although both CD4^+^ T_EM_ and T_CM_ populations produced IFN-γ, CD4^+^ T_CM_ cells were predominantly integrin α_4_β_7_^+^ while CD4^+^ T_EM_ were found to include both integrin α4β7^+^ and integrin α4β7^−^ cells. In contrast, IFN-γ-producing CD8^+^ cells were predominantly classical T_EM_ and CD45RA^+^ T_EM_ (T_EMRA_; CD45RO^−^ CD62L^−^) subsets. Interestingly, while CD8^+^ T_EM_ included both integrin α4β7^+^ and integrin α4β7^−^ cells, CD8^+^ T_EMRA_ were predominantly integrin α_4_β_7_^+^ (152). By using PBMC from healthy adults immunized with the Ty21a vaccine, we have also reported that *S*. Typhi-specific CD8^+^ T-cells are able to co-express high levels of integrin α_4_β_7_, intermediate levels of CCR9 and low levels of CD103 (83). Furthermore, we showed that these specific memory CD8^+^ T-cells with gut homing potential bear multiple TCR Vβ specificities (e.g., Vβ2, 3, 8, 14, and 17) (83). Of note, cells used in this study were collected 5–40 months after oral immunization. Thus, *S*. Typhi-specific CD8^+^ T_EM_ cells with gut homing potential might persist in circulation over long periods of time. However, because the study used cells isolated exclusively from peripheral blood, we have to consider the possibility that these observations might not reflect the full spectrum of TCR Vβ usage by *S*. Typhi-specific CD8^+^ T-cells in the gut microenvironment *in vivo*. Based on these findings regarding the homing potential of *S*. Typhi-specific cells, it is reasonable to speculate that the observed multiphasic kinetics of the T-cell responses described above might represent decreases in circulating *S*. Typhi-specific T-cells as they home to the gut and other lymphoid tissues, as well as increases due to the release into the circulation of new waves of specific cells generated in lymphoid organs.

## Microbiota, Co-Infections, and the Host Immune Response Following Immunization with Oral Attenuated Typhoid and Other Enteric Vaccines

There is growing evidence from clinical studies indicating that the gut microbiota has a profound impact in modulating human immune responses in health and disease, including a significant role in influencing vaccine efficacy (190, 211–213). For example, in a study evaluating the oral attenuated *V. cholerae* O1 vaccine CVD 103-HgR, Lagos and colleagues demonstrated that excessive bacterial growth (“tropical enteropathy”) in the small intestine of children in less developed countries might contribute to the low-antibody response to the vaccine (214). In this study, an inverse association was found between bacterial over growth and seroconversion as determined by vibriocidal titers. Reduced vaccine efficacy and immunogenicity in developing countries when compared with North Americans also has been reported with other vaccines, including oral polio and rotavirus (137, 213, 215). Helminth infections have also been demonstrated to impact vaccine immunogenicity and, for example, anti-helminthic therapy prior to immunization was shown to improve the immune response to the CVD 103-HgR cholera vaccine (216). Regarding *S*. Typhi, recent evidence showed that the induction of *S*. Typhi-specific IgG LPS antibodies following immunization was significantly higher among CVD 908-*htrA* vaccines infected with *Helicobacter pylori* than in uninfected subjects. These results are likely the consequence of gastric acid hyposecretion due to *H. pylori* infection which facilitated the passage of CVD 908*-htrA* through the stomach (217). These observations are supported by reports indicating that the risk of developing typhoid fever is higher in *H. pylori*-infected individuals in underdeveloped countries (218), suggesting that the success of the Ty21a typhoid vaccine in endemic regions might be the result, at least in part, of the high prevalence of *H. pylori* infection accompanied by hypochlorhydria (217, 219). Additionally, evidence in animal models suggests that modulation of the gut microbiota (e.g., with antibiotics, prebiotics, and probiotics) can enhance vaccine efficacy (220, 221).

We recently initiated studies to directly investigate the interactions between the microbiome and vaccination with attenuated oral vaccines. We observed that, although Ty21a is a live-attenuated *S*. Typhi vaccine delivered via the oral route, there was no disruption in the composition, diversity, or stability of the fecal microbiota in healthy adult volunteers who received this vaccine (172). However, categorical analysis based on multiphasic CMI responses versus late CMI responses identified a subset of bacterial operational taxonomic units (OTUs) differentiating individuals capable of mounting distinct immunological responses. Generally, individuals who exhibited a multiphasic CMI response to vaccination harbored greater community richness and diversity compared to individuals with only a late CMI response to Ty21a. No differences were identified in community richness or diversity among volunteers characterized as responders or non-responders based on seroconversion (*S*. Typhi LPS). Although the number of volunteers analyzed was small, this study provides additional information supporting the potential influence of the gut microbiota on the immune response elicited by oral immunization, and perhaps, in protection. Additional studies involving larger numbers of volunteers and a multiplicity of vaccines administered via the oral route are necessary to extend our understanding of the complex role of the gut microbiota in modulating host immunity and vaccination in humans, and its possible role in vaccine efficacy.

## Cross-Reactive Immune Responses Among *S*. Typhi, *S*. Paratyphi A, and *S*. Paratyphi B

As discussed above, limited information is available regarding host immune responses to *S*. Paratyphi A and *S*. Paratyphi B in humans. In fact, most of the immune responses believed to be elicited by *S*. Paratyphi A have been inferred from *S*. Typhi studies. Interestingly, field trials of Ty21a have shown modest cross-protection against *S*. Paratyphi B (3), suggesting that cross-reactive immune responses might be responsible. The presence of cross-reactive responses were first reported in the 1980s by Tagliabue et al. who reported the induction of IgA antibodies following oral immunization with Ty21a, which mediate T-cell-dependent ADCC against *S*. Typhi, *S*. Paratyphi A, and *S*. Paratyphi B, but not against *S*. Paratyphi C (87). We have recently identified cross-reactive immunological responses against *S*. Paratyphi A and *S*. Paratyphi B in subjects orally immunized with Ty21a (91). IgA ASC that recognized LPS from *S*. Paratyphi A and *S*. Paratyphi B were observed, but at a lower magnitude than responses against *S*. Typhi LPS (91). These cross-reactive anti-LPS CD19^+^ CD27^+^ IgG and IgA ASC displayed the same homing pattern (i.e., a dominant integrin α_4_β_7_^+^ CD62L^−^subset and a significant proportion of integrin α_4_β_7_^+^ CD62L^+^ cells) as *S*. Typhi-specific ASC. We also reported the induction of antibodies and B_M_ to *S*. Typhi LPS and OMP antigens, which cross-react with *S*. Paratyphi A and *S*. Paratyphi B. However, IgA B_M_ reactive to *S*. Typhi was of higher magnitude than those against *S*. Paratyphi A and *S*. Paratyphi B. In contrast, B_M_ to outer membrane proteins (OMP) from *S*. Paratyphi B were similar to those observed for *S*. Typhi-OMP, but higher than those for *S*. Paratyphi A OMP. In a subsequent study, we reported in Ty21a and CVD 909 vaccines the presence of cross-reactive serum antibodies able to mediate opsonophagocytosis of *S*. Paratyphi A and *S*. Paratyphi B, albeit at lower levels than those against *S*. Typhi (90, 91). Similar observations regarding cross-reactive ASC responses among *S*. Typhi and *S*. Paratyphi serovars A, B, and C were recently reported in Ty21a vaccinees and patients with enteric fevers (222). These cross-reactive responses are likely the result of the immunity elicited by O:12, the trisaccharide (mannose–rhamnose–galactose) repeating unit that comprises the LPS backbone, which is common to *S*. Typhi, *S*. Paratyphi A, and *S*. Paratyphi B. Of note, a recent study showed that, although *S*. Paratyphi A and *S*. Paratyphi B do not possess the Vi antigen, cross-reactive ASC were identified in recipients of the Vi polysaccharide vaccine (223). The authors concluded that this low level of cross-reactivity is likely attributable to *S*. Typhi-LPS contamination of the Vi polysaccharide vaccine. Similar observations were reported by others (89, 139). Of note, although to our knowledge there are no reports documenting cross-protection against non-typhoidal *Salmonella* in Ty21a or Vi vaccinees, these typhoid immunizations elicit cross-reactive ASC against non-typhoidal *Salmonella*, including *S*. Typhimurium and Enteriditis that share either O:9, O:12, or both antigens with *S*. Typhi (224, 225). In spite of these studies, the precise immune mechanism(s) of the cross-protection observed against *S*. Paratyphi B in Ty21a vaccinees in field trials remains unclear. However, it is tempting to speculate that CMI responses might play a key role in cross-protection. Further studies assessing the basis for these cross-reactive responses, as well as whether immunization with novel attenuated *S*. Paratyphi A vaccines, or wild-type *S*. Paratyphi A infection, results in cross-reactive humoral and CMI responses with *S*. Typhi and *S*. Paratyphi B will provide critical information to advance the development of broad-spectrum vaccines to protect against enteric fevers.

## “Omics” Studies

Recent advances in microarray and proteomics technologies have allowed for detection of immunogenic *S*. Typhi antigens (226, 227). Both immunoaffinity proteomics-based technology and protein microarrays have been utilized to identify key antigens that may be suitable for vaccine development and diagnostics (226, 227). Furthermore, transcriptional profiling in peripheral blood of patients infected with *S*. Typhi identified a distinct and reproducible signature that changed during treatment and convalescence (228). Additionally, studies performed in mice and humans have also identified immune signatures common to murine and human systemic salmonellosis (229). Although very few manuscripts have reported the use of these state-of-the-art approaches, these comprehensive analyses of the transcriptional and proteomic profiles provide a foundation for more directed analyses that may have a direct impact on the development of novel vaccines and diagnostics in coming years.

## Concluding Remarks

Despite decades of effort, the mechanisms of protective immunity in natural infection and vaccination remain largely undefined and many questions remain (Box 1). The vast majority of the information currently available using modern immunological techniques has been obtained using specimens from subjects immunized with attenuated typhoid vaccines. Old challenge studies lacked the appropriate tools to monitor immune cells (e.g., B- and T-cells) and in general, have been limited to measurements of serum antibody titers and, in some cases, the use of inadequate CMI methodology available at that time. The “Renaissance” of challenge studies with wild-type *S*. Typhi, such as those being performed in Oxford, is at hand and novel technologies to analyze in unprecedented depth the host immune responses have recently become available. One of these technologies is mass cytometry, also known as Cytometry by Time Of Flight (CyTOF), capable of resolving more than 35 measurements per cells using rare metal-conjugated monoclonal antibodies with minimal signal overlap (230–232); a problem that severely limits the number of parameters, which can be evaluated by conventional flow cytometry. This novel technology will enable the simultaneous measurement of the phenotype and function of multiple immune cell types by simultaneously monitoring the cross-talk between traditional players (e.g., B- and T-cells), and new potential players (e.g., innate-like T-cells, including TCRγδ T-cells, NK-T-cells, and MAIT cells, as well as T_reg_ cells) and the possible mechanisms leading to protection against infection. In fact, it is likely that it is the balance (i.e., homeostasis) between effector and regulatory responses that holds the key to understanding protective immunity. Mass cytometry, in conjunction with traditional immunological assays and state-of-the-art genomics, transcriptomics, proteomics, and metabolomics approaches and the availability of human challenge models provide, for the first time, the necessary tools to uncover the mechanisms underlying protective immunity, both systemically and in the gut microenvironment. This information will be invaluable in accelerating the development of novel vaccine strategies to prevent enteric fevers. In addition, the expected explosion of knowledge regarding the gut microbiome and its role in modulating immunity to oral vaccines is also likely to provide significant insights in coming years in understanding the observed differences in immunogenicity between vaccine responses in developed and developing countries.

Box 1 **Key remaining questions.**

- What are the relative contributions of humoral and cellular responses to protection?
- What are the precise roles of effector and memory B and T-cells, as well as innate immune cells in protection?
- How can an appropriate balance between pro-inflammatory and regulatory responses be achieved, resulting in protection without causing excessive inflammation?
- What are the mechanisms of enduring protection against *S*. Typhi, *S*. Paratyphi A, and *S*. Paratyphi B and how can long-lasting responses be preferentially induced?
- What are the characteristics of protective local gut immune responses?
- What are the differences and similarities between local and systemic immune responses?
- What is the role of the gut microbiota in modulating immune responses against enteric fevers?
- Can cross-reactive immune responses between *S*. Typhi, *S*. Paratyphi A, and *S*. Paratyphi B be exploited to develop broad-spectrum vaccines against enteric fevers?

## Conflict of Interest Statement

The authors declare that the research was conducted in the absence of any commercial or financial relationships that could be construed as a potential conflict of interest.
